# An Analytical Comparison Between Ketamine Alone and a Combination of Ketamine and Propofol (Ketofol) for Procedural Sedation and Analgesia From an Emergency Perspective: A Systematic Review and Meta-Analysis

**DOI:** 10.7759/cureus.27318

**Published:** 2022-07-26

**Authors:** Hany A Zaki, Nabil Shallik, Eman Shaban, Khalid Bashir, Haris Iftikhar, Yousra Mohamed Khair, Mohammed Gafar Abdelrahim, Mohamed Fayed, Mohamed Hendy, Emad El-Din Salem, Amr Elmoheen

**Affiliations:** 1 Emergency Medicine, Hamad Medical Corporation, Doha, QAT; 2 Clinical Anesthesia, Weill Cornell Medical College - Qatar, Doha, QAT; 3 Clinical Anesthesia, Qatar University, Doha, QAT; 4 Clinical Anesthesia and Surgical Intensive Care Unit (SICU), Tanta University Faculty of Medicine, Tanta, EGY; 5 Anesthesia, Hamad Medical Corporation, Doha, QAT; 6 Cardiology, Al Jufairi Diagnostic and Treatment, Doha, QAT; 7 Medicine, Qatar University, Doha, QAT; 8 Medical Education and Simulation, Weill Cornell Medical College - Qatar, Doha, QAT; 9 Accident and Emergency, Hamad Medical Corporation, Doha, QAT; 10 Anesthesia, Armed Forces Hospitals, Cairo, EGY

**Keywords:** systematic review, meta-analysis, emergency medicine, procedural sedation and analgesia, ketofol, ketamine, propofol

## Abstract

Procedural sedation and analgesia (PSA) is a treatment approach involving treating patients with agents with dissociative, sedative, or analgesic properties to suppress their consciousness to variable levels. Ketamine and propofol have been used historically for PSA. Because they each have their demerits, it was postulated that combining both drugs (ketofol) would result in a mixture with additive properties and lessen or eliminate the demerits attributed to each drug. The primary objective of this systematic review and meta-analysis is to compare ketamine alone and a combination of ketamine and propofol (ketofol) for procedural sedation and analgesia from an emergency perspective.

A systematic search was conducted on published studies from the databases of Scopus, ScienceDirect, PubMed, Google Scholar, APA PsycInfo, and the Cochrane Central Register of Controlled Trial (CENTRAL) until July 2022. The articles that were published on the online databases were authored between January 2007 and 2018. The selected papers were scanned and examined to check whether they met the eligibility criteria for the study.

The search produced six articles that were included in the systematic review and meta-analysis. All six articles that passed the eligibility criteria were viable for the analysis. All the trials focused on the effectiveness of ketofol versus ketamine for PSA from an emergency perspective.

Ketofol was found to be safe and more effective in comparison to ketamine for PTA.

## Introduction and background

In many cases, patients require procedural sedation and analgesia (PSA), especially those in the emergency department (ED) that undergo significantly painful medical procedures. PSA is mainly applied in patients undergoing procedures such as cardioversion, reduction of body fractures, and abscess drainage. The objective of PSA for patients is to improve the procedure’s success rate, overall comfort, and medical satisfaction [1]. Propofol is a hypnotic or sedative often used for procedural sedation; it offers numerous medical advantages, including fastidious impact on the patient, titration ease, and prompt action onset compared to other pharmacological treatments [2]. Unfortunately, propofol use has some detrimental implications on the patients in some cases, such as hemodynamic compromise and respiratory depression; most of these effects depend on the dosage quantities. Additionally, according to a previous study, ketamine use is more effective when its main emphasis is to sedate the individual as fast as possible; however, it has some negative implications that must be considered [3]. Fortunately, despite the fact that the drug has been proven to have antiemetic and amnesiac effects, it is not analgesic [4].

For purposes of pain reduction for patients, other drugs and components are included. An appropriate agent for procedural sedation and analgesia should conventionally have no adverse implications, offer brief periods of recovery, remain predictable and consistent in effect, and have a swift onset. Considering that, to date, no single drug has been able to meet all the aforementioned expectations, it is best to identify and combine drugs with significant strengths [1]. A combination or mixture of different dissociatives, sedatives, or analgesics may be quite advantageous. Researchers, experts, and scientists have previously proposed that the combination of propofol and ketamine (ketofol) mixed in one could be quite effective [5]. In the aforementioned case, procedural sedation allows for a decrement in the required dosage of each specific agent that forms the mixture, which potentially alleviates the earlier mentioned risk of adverse respiratory implications associated with the use of propofol solely or a mixture with opioids or other components [4]. Additionally, ketamine addition may potentially reduce the hemodynamic instability risk while providing analgesia and the required level of sedation [6].

Unfortunately, the use of ketamine solely may result in the patient experiencing vomiting or nausea and post-procedural agitation in many cases. Therefore, the two components, propofol and ketamine, each have negative properties. The mixture of the two agents can be prepared in either completely separate syringes or in one syringe mixture. According to previous research, the mixture, ketofol, has been found to have physical compatibility and chemical stability when in polypropylene syringes. The central objective of the current systematic review and meta-analysis was to compare ketamine alone and a combination of ketamine and propofol (ketofol) for procedural sedation and analgesia from an emergency perspective.

## Review

Search methods

Search Criteria and Information Sources

This research paper was reported following the Preferred Reporting Items for Systematic Reviews and Meta-Analyses (PRISMA) guidelines. Online databases were searched for articles, including Scopus, ScienceDirect, PubMed, Google Scholar, APA PsycInfo, and the Cochrane Central Register of Controlled Trial (CENTRAL). Table 1 shows the search string applied for some of the online databases.

**Table 1:** Search strings and keywords for online database search

Search	Search string
#1	“Ketamine”
#2	“Propofol”
#3	“Ketofol”
#4	(“Ketamine” OR “Propofol”) AND (“Procedural Sedation and Analgesia”) in Title, Abstract, and Keywords
#5	(“Ketamine” OR “Propofol” OR “Ketofol”) AND (“Procedural Sedation and Analgesia”) in Title, Abstract, and Keywords

The keywords applied for the search include “Ketamine,” “Propofol,” ‘‘Ketofol,” “Procedural Sedation and Analgesia,” and “Emergency.” All articles were assessed to gauge their relevance to the research topic and were published through conventional channels.

Inclusion Criteria

All the included articles were published in the English language. As expected, relevance to the research topic was the most significant inclusion criterion. All articles were authored between 1990 and 2022. Only peer-reviewed journal articles with accessible full texts and abstracts were considered for inclusion. Participants included in the studies must have undergone mildly painful procedures, including but not limited to orthopedic manipulation, burn-dressing procedures, wound debridement, dislocation, electrical cardioversion, mild body fractures, laceration suturing, or procedures to eliminate foreign material from the body. All articles had to be published through the correct channels.

Exclusion Criteria

Any studies authored in languages other than English or published earlier than 1990 were excluded from the analysis. Incomplete trials or unpublished scholarly work were not included. Additionally, studies irrelevant to the research topic were excluded. Sources that included participants that had undergone extreme surgeries were excluded. Studies that included pregnant women were also excluded. Grey literature, case studies, and non-journal papers were also not included. Any sources irrelevant to the research topic were excluded. Studies that were relevant to the research topic but had small sample sizes in single-agent analyses were excluded in the current analysis because a non-usable format failed to support the generation of a suitable and meaningful conclusion. Studies with less than 50 participants were excluded.

Statistical Analysis

The Review Manager was used for the analysis of the implications of using ketamine alone or a combination of ketamine and propofol; the study was conducted and interpreted according to the Cochrane Handbook. Pooled prevalence for each side effect was also calculated. A fixed-effects model was used, alongside a confidence interval (CI) of 95%. The heterogeneity between studies was assessed using the I2 statistics, and a P-value of 0.05 was considered the significance threshold.

Search results

**Figure 1:**
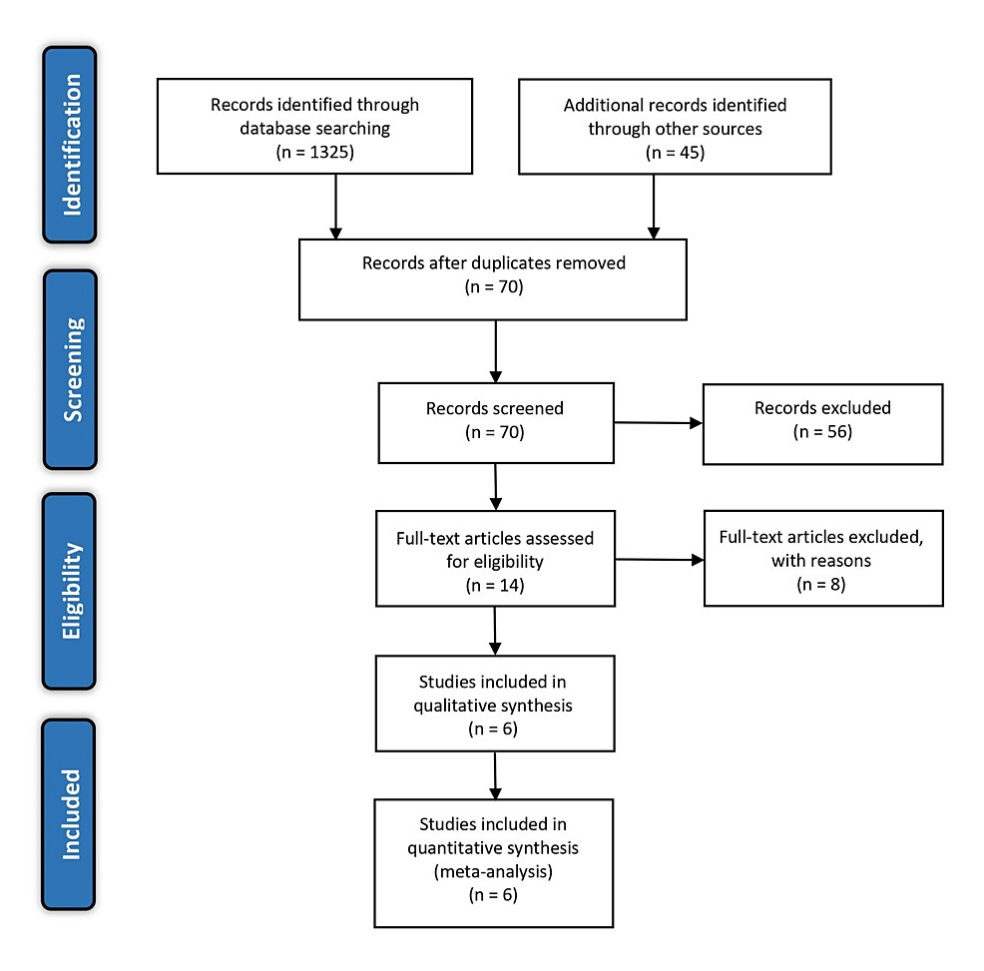
PRISMA flow diagram of the literature search results PRISMA: Preferred Reporting Items for Systematic Reviews and Meta-Analyses

A total of 1,325 articles were identified from database search and 45 from Google Scholar and screening of reference lists. After removing duplicates, 70 articles remained. When the titles and abstracts of the 70 non-duplicate sources were read and carefully screened, 56 articles were excluded as they failed to directly report on the comparison between ketamine alone and a combination of ketamine and propofol (ketofol) for procedural sedation and analgesia from an emergency perspective. The remaining 14 articles were full-text articles, but only six of them were fully relevant to the research topic and passed the inclusion criteria. Figure 1 shows the study selection process following the PRISMA guidelines.

Results

Ketofol in the Emergency Department

Willman and Andolfatto (2007) [7]: Very few studies compare the effectiveness of ketofol to ketamine for procedural sedation and analgesia (PSA) in an emergency department (ED). The prospective study of Willman and Andolfatto looked at ketofol effectiveness for PSA conducted in an ED setting [7]. In the study, 114 patients who required PSA primarily for orthopedic procedures were selected. They were given ketofol, which included a 1:1 ratio mixture of ketamine (10 mg/mL) and the same quantity for propofol in addition to 1-3 mL aliquots that were titrated at the treating physician’s discretion. The researchers recorded the initial dose administered to the participants of the study, procedural success, vital signs at predefined intermissions, adverse events’ absence or presence, recovery period, and patient or physician satisfaction. The average medication dosage administered was approximately 0.75 mg/kg for propofol and the same for ketamine. Fortunately, none of the participants were hypotensive, and there was no proof or sign of poor perfusion. A significant finding of the prospective study was that 2.6% of the participants (95% CI: 0.6%-7.5%) had transient hypoxia, and among them, 0.9% (95% CI: 0.02%-4.8%) needed bag valve mask (BVM) ventilation. None of the participants experienced aspiration or vomiting. Three (2.6%) of the 114 participants (95% CI: 0.6%-7.5%) experienced an emergency reaction to the ketofol administered, of which one was given midazolam. The study showed a procedural success rate of 96.5% without the integration of adjunctive medications. The median time until the participants fully recovered was found to be approximately 15 minutes (ranging from five to 45 minutes).

Yalcin et al. (2018) [8]: Seventy-five American Society of Anesthesiologists (ASA) class I patients were randomly selected and included in the trial. The participants between the ages of six and 12 were diagnosed with high levels of anxiety. They were divided into three groups: group 1 (1 mg/kg dosage of ketamine), group 2 (2 mg/kg dosage of propofol), and group 3 (0.6 mg/kg dosage of ketamine plus propofol (ketofol)), followed by 40-60 µg/kg/minute continuous in fusion) [8]. According to the findings of the trial, there was a greater rate of complication in the first group (those treated with ketamine) (P<0.05). Additionally, the average recovery time was found to be statistically shorter in the ketofol group in comparison to the ketamine-treated group (P<0.05). Both ketamine plus propofol and propofol groups were also found to have similar associations between the levels of sedation and the bispectral index (BIS) values. In contrast, there was no relationship between the two aforementioned aspects in the group treated using ketamine. Ultimately, the anxiety levels of children in the group treated with ketofol decreased significantly compared to the ketamine-treated group (P<0.05).

Andolfatto and Willman (2011) [9]: This prospective study, similar to the previous case, assessed the effectiveness of ketofol when used for PSA. It was found to be as effective in 98% (n=717) of the participants. Ketofol was used for PSA in 728 participants, mainly for orthopedic treatments. As shown in Table 2, the median patient age was 53 years (ranging from 21 to 99 years, with interquartile range (IQR) ranging from 36 to 70 years). The median dosage quantity of ketamine and propofol was 0.7 mg/kg each (range: 0.2-2.7 mg/kg; IQR: 0.5-0.9 mg/kg), and the median recovery time was 14 minutes (range: 3-50 minutes; IQR: 10-17 minutes). PSA was effective in 717 (98%) cases. Bag mask ventilation occurred in 15 (2.1%) patients (95% CI: 1%-3.1%). One participant experienced vomiting, and another was admitted to the medical institution for monitoring of both hypotensions and transient dysrhythmia. No sequelae were identified. The average satisfaction scores of the medical personnel, including nurses and physicians, were 10 (IQR: 9-10) on a scale of 1 to 10. Owing to the effectiveness of ketofol, 97% of the participants accepted that they would have chosen the same PSA method in the future. The mixture was not effective in 11 of the participants, of which nine of them were successfully treated using propofol. In 2/9 unsuccessful cases, the procedure had to be terminated as a function of inadequate sedation. The two participants were undergoing attempted shoulder dislocation reduction; in the two cases, the PSA procedure was not completed due to muscular rigidity. There is a limitation and deficiency in the literature review for article documentation regarding ketofol use in the emergency department [10,11].

Shah et al. (2011) [12]: A total of 167 participants took part in this trial, of which 67 were treated with ketamine/propofol, while 69 were treated with ketamine. The median time of sedation was longer in the case of ketamine (approximately 16 minutes) in comparison to ketofol (approximately 13 minutes) (Δ: -3 minutes; 95% CI: -5 to -2 minutes). Additionally, the median time for recovery taken was slower in the case of ketamine (approximately 12 minutes) and shorter in the case of ketamine/propofol (approximately 10 minutes) (Δ: -2 minutes; 95% CI: -4 to -1 minute). Although there were cases of vomiting in both groups, there was more vomiting/nausea in the ketamine group (12% of the participants) compared to the ketamine/propofol group (2% of the participants) (Δ: -10%; 95% CI: -18% to -2%). The satisfaction scores were much greater in the ketofol group (P<0.05).

Weisz et al. (2017) [13]: Ninety-six participants were randomized into the ketamine group, and 87 patients were randomized to coadministration of ketamine and propofol. According to the primary findings, there was no significant difference in type/nature or extent of the adverse events, excluding nausea, which was found to be more prevalent in the ketamine group. Additionally, the PSA efficacy was greater in the ketamine group (99%) in comparison to the ketofol group (90%). Unlike other previous studies, the median recovery time was similar for both the ketamine and ketofol groups. Additionally, the scores measuring satisfaction by medical personnel, including physicians and nurses, were greater for the ketofol group; however, the patients were equally satisfied with both regimens of sedation. The adverse implications were documented more regularly when observed by an autonomous party rather than conveyed by healthcare providers caring for the participants [14].

Aboeldahab et al. (2011) [15]: The study compared three: groups P (propofol), K (ketamine), and KP (ketofol). Since the current systematic review and meta-analysis focuses on ketofol and ketamine comparison, the propofol findings will not be discussed. According to the findings, the time required for verbal contact loss and the reflex of eyelashes was found to be much earlier in the ketofol group in comparison to the ketamine group, and the difference between the two cases was statistically significant. Following induction, the mean arterial blood pressure in the ketofol group was fairly comparable to baseline, while it increased in the ketamine group; the difference in the two cases was quite significant. The mean arterial blood pressure following intubation was significantly higher in the ketamine group. Additionally, following induction, similar to the mean arterial blood pressure, the heart rate measured was found to be comparable to baseline in the ketofol group and increased in the ketamine group; the difference between the two cases was statistically significant. After a while, heart rate decreased in both the ketofol and ketamine groups and afterward became stable and comparable during the rest of the surgical time.

Data Extraction Results

Table 2 presents descriptions of the included studies.

**Table 2:** Study descriptor table BVM: bag valve mask

Author and year	Study design	Study characteristics	Participant characteristics	Population
Willman and Andolfatto (2007) [7]	Prospective study	Ketofol (0.75 mg/kg of ketamine and 0.75 mg/kg of propofol)	Emergency department (no cases of hypotension, one of the patients required BVM ventilation, and three patients had an emergency reaction)	114 adults
Yalcin et al. (2018) [8]	Randomized controlled trial	Ketofol versus ketamine versus propofol: group 1 (ratio: 1:1; 200 mg propofol (20 mL) + 200 mg ketamine (4 mL), group 2 (4 mL ketamine diluted with NS to 20 mL, 1 mg/kg bolus followed by 50-60 μg/kg/minute), and group 3 (2 mg/kg propofol bolus followed by 70-90 μg/kg/minute infusion)	Age: 6-12 years; ailment: dental treatment	75 children
Andolfatto and Willman (2011) [9]	Prospective study	Ketofol (the median dose of medication used was 0.7 mg/kg)	Median patient age: 53 years	728 adults received ketofol
Shah et al. (2011) [12]	Randomized controlled trial	Ketofol versus ketamine: group 1 (0.5 mg/kg ketamine + 0.5 mg/kg propofol), group 2 (1 mg/kg ketamine + intralipid placebo)	Children (7-14 years), closed manual reduction	136 children
Weisz et al. (2017) [13]	Randomized, single-blinded, controlled trial	Ketofol versus ketamine: group 1 (0.5 mg/kg ketamine and 0.5 mg/kg propofol), group 2 (1 mg/kg ketamine)	Age: (ketofol: mean (SD): 9.3 (5); ketamine: mean (SD): 8.3 (6)); ailment: reduction of fracture of dislocation	183 children
Aboeldahab et al. (2011) [15]	Comparative study	Group K: intravenous ketamine in a dose of 2 mg/kg over 20 s; group P: intravenous propofol 1% in a dose of 2 mg/kg over 20 s; group KP: intravenous ketofol, prepared in a ratio of 1:1 as follows: 100 mg ketamine + 100 mg propofol	20-50 years old, ASA physical status I and II, patients had no history of cardiovascular or neurologic disease undergoing hernia repair operations	60 adult participants

Statistical Analysis

**Figure 2:**
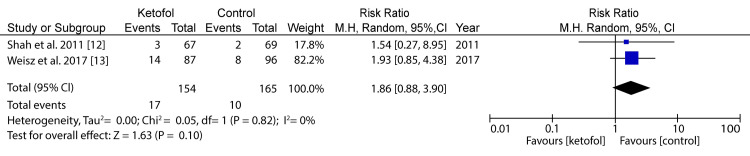
Forest plot comparing ketofol versus ketamine with respect to desaturation Shah et al. (2011) [12] and Weisz et al. (2017) [13]

Figure 2 shows a forest plot comparing ketofol versus ketamine as regards desaturation.

**Figure 3:**
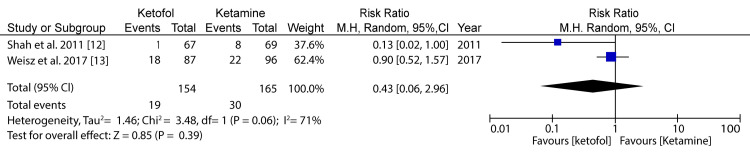
Forest plot comparing ketofol versus ketamine with respect to vomiting Shah et al. (2011) [12] and Weisz et al. (2017) [13]

In the analysis of desaturation in the case of ketofol versus ketamine as one of the primary outcomes, two randomized controlled trials (RCTs) were included for analysis. The minimum population was 67 participants, and the maximum was 96 participants; for the two studies, the total number of participants was 154 for the study group and 165 for the control group. A random-effects model was applied for the subgroup analysis. The difference in the primary outcome for desaturation in the case of ketofol versus ketamine as the control was 1.86 (95%CI: 0.88-3.9) on a 0-10 visual analog scale (VAS), favoring the treatment using ketamine. The difference between the two cases was statistically insignificant (P=0.1), which was below the P-value threshold of P=0.05. The selected articles had very low heterogeneity (P<0.00), and the I2 statistic is equal to 0%. Therefore, there was no significant difference in terms of desaturation between the two groups. Another forest plot comparing ketofol versus ketamine as regards vomiting is shown in Figure 3.

**Figure 4:**
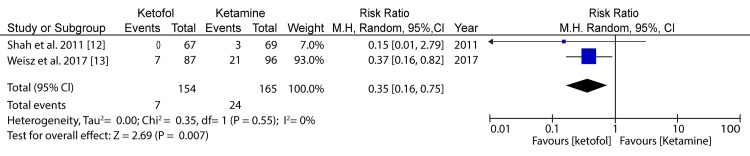
Forest plot comparing ketofol versus single-agent control with respect to nausea Shah et al. (2011) [12] and Weisz et al. (2017) [13]

In the analysis of vomiting in the case of ketofol versus ketamine as one of the primary outcomes, two RCTs were included for analysis. The minimum population was 136 participants, and the maximum was 183 participants; for the two studies, the total number of participants was 154 for the study group and 165 for the control group. A random-effects model was applied for the subgroup analysis. The difference in the primary outcome for vomiting in the case of ketofol versus ketamine as the control was 0.43 (95%CI: 0.06-2.96) on a 0-10 visual analog scale (VAS), favoring the treatment using ketofol. The difference between the two cases was statistically insignificant (P=0.06), which was below the P-value threshold of P=0.05. The selected articles had very low heterogeneity (P<0.00), and the I2 statistic is equal to 0%. Therefore, there was a slightly significant difference in terms of vomiting between the ketofol and ketamine groups. Figure 4 shows a comparison of ketofol versus ketamine with regard to nausea.

**Figure 5:**
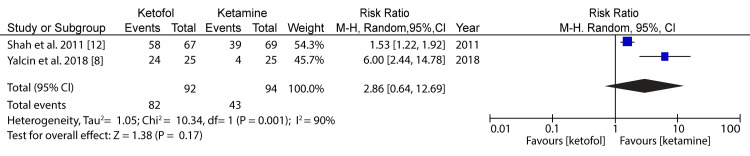
Forest plot comparing ketofol versus ketamine with respect to clinical satisfaction Shah et al. (2011) [12] and Yalcin et al. (2018) [8]

In the analysis of nausea in the case of ketofol versus ketamine as one of the primary outcomes, two RCTs were included for analysis (Shah et al. (2011) [12] and Weisz et al. (2017) [13]). The minimum population was 136 participants, and the maximum was 183 participants; for the two studies, the total number of participants was 154 for the study group and 165 for the control group. A random-effects model was applied for the subgroup analysis. The difference in the primary outcome for vomiting in the case of ketofol versus ketamine as the control was 0.35 (95%CI: 0.16-0.75) on a 0-10 visual analog scale (VAS), favoring the treatment using ketofol. The difference between the two cases was statistically insignificant (P=0.007), which was below the P-value threshold of P=0.05. The selected articles had very low heterogeneity (P<0.00), and the I2 statistic is equal to 0%. Therefore, there was a significant difference in terms of nausea between the ketofol and ketamine groups. Figure 5 shows a comparison of ketofol versus ketamine with regard to clinical satisfaction.

**Figure 6:**
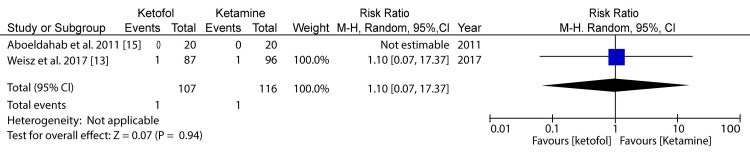
Forest plot comparing ketofol versus ketamine with respect to apnea Aboeldahab et al. (2011) [15] and Weisz et al. (2017) [13]

In the analysis of clinical satisfaction in the case of ketofol versus ketamine as one of the primary outcomes, two RCTs were included the analysis (Shah et al. (2011) [12] and Yalcin et al. (2018) [8]). The minimum population was 136 participants, and the maximum was 50 participants; for the two studies, the total number of participants was 92 for the study group and 94 for the control group. A random-effects model was applied for the subgroup analysis. The difference in the primary outcome for clinical satisfaction in the case of ketofol versus ketamine as the control was 2.86 (95%CI: 0.64-12.69) on a 0-10 visual analog scale (VAS), favoring the treatment using ketamine. The difference between the two cases was statistically significant (P=0.17), which was above the P-value threshold of P=0.05. The selected articles had very low heterogeneity (P<0.001), and the I2 statistic is equal to 90%. Therefore, there was a significant difference in terms of clinical satisfaction between the ketofol and ketamine groups. Figure 6 shows a comparison of ketofol versus ketamine with respect to apnea.

In comparing ketofol versus ketamine with respect to apnea, one of the primary outcomes, two RCTs were included for analysis. The minimum population was 40 participants, and the maximum was 183 participants; for the two studies, the total number of participants was 92 for the study group and 116 for the control group. A random-effects model was applied for the subgroup analysis. The difference in the primary outcome for apnea in the case of ketofol versus ketamine as the control was 1.10 (95%CI: 0.07-17.37) on a 0-10 visual analog scale (VAS), favoring the treatment using ketamine. The difference between the two cases was statistically significant (P=0.07), which was above the P-value threshold of P=0.05. The heterogeneity and I2 statistic were not applicable. Therefore, there was a very insignificant difference in terms of apnea between the ketofol and ketamine groups.

Risk of Bias

**Figure 7:**
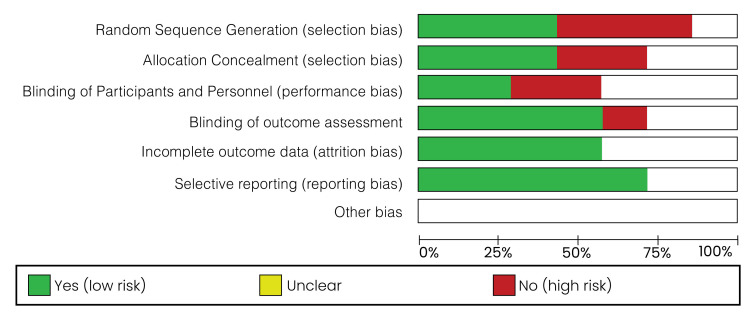
Risk of bias graph: a review of authors’ judgments about each risk of bias item presented as percentages across all included studies

**Figure 8:**
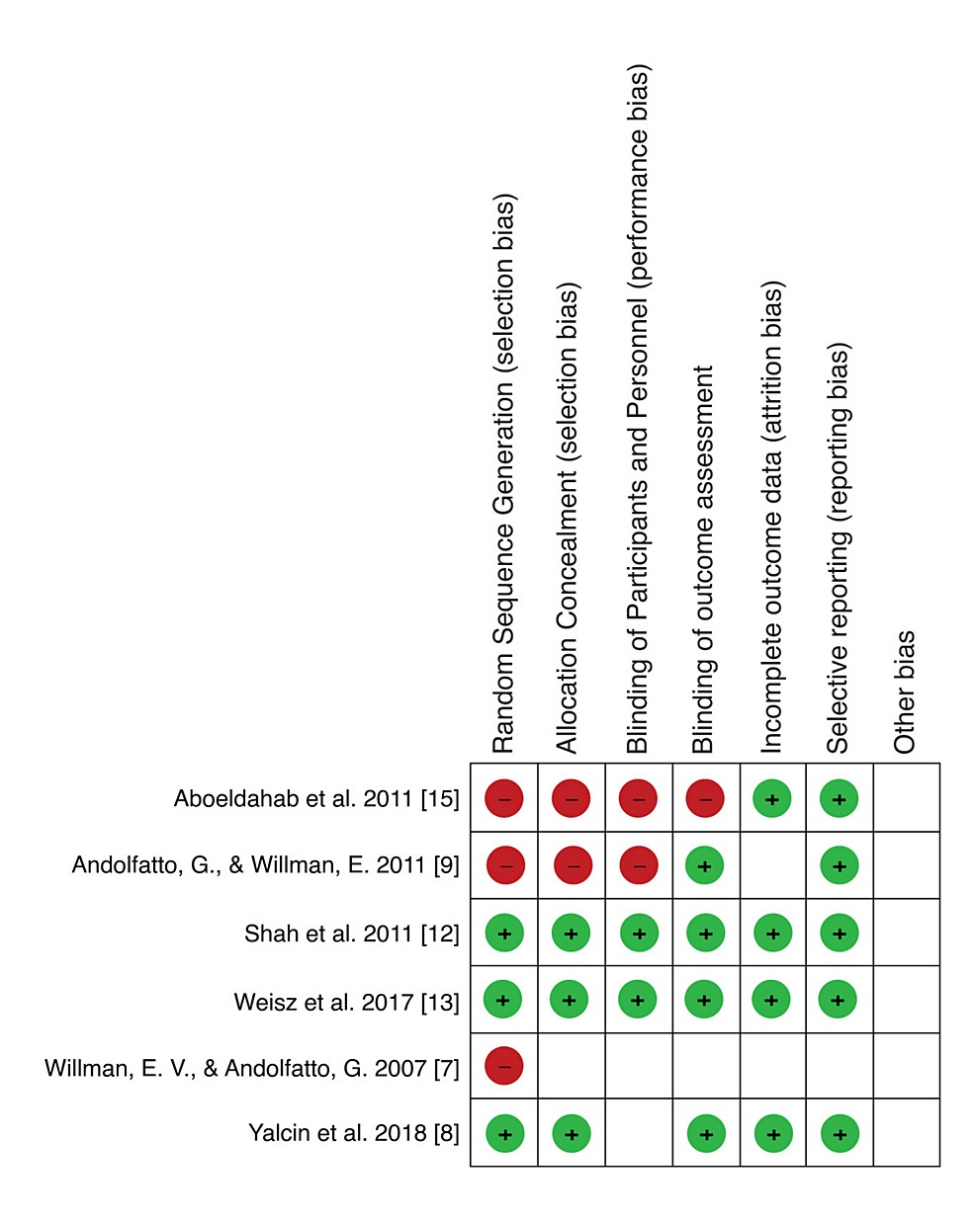
Risk of bias summary: a review of authors’ judgments about each risk of bias item for each included study Aboeldahab et al. (2011) [15], Andolfatto and Willman (2011) [9], Shah et al. (2011) [12], Weisz et al. (2017) [13], Willman and Andolfatto (2007) [7], and Yalcin et al. (2018) [8]

The risk of bias graph is shown and illustrated in Figure 7, and the risk of bias summary is shown and illustrated in Figure 8.

The parameters of risk of bias were evaluated, including random sequence generation, allocation concealment (selection bias), blinding of participants and personnel, blinding of outcome assessment, completeness of data, selectivity of outcome reporting, and other biases. According to the risk of bias summary (Figure 8), there was a high risk of bias, especially in relation to randomization. The risk in relation to other biases was unclear. Generally, the risk of bias was found to be low. After performing the risk of bias assessment independently, any disagreements were resolved successfully through discussion.

Discussion

The concept of the mixture of ketamine and propofol to form ketofol is founded on the synergistic implications and the advantages they provide while countering the effects of each of the two components. The primary objective of the current systematic review and meta-analysis was to analyze the effectiveness of ketofol (ketamine and propofol) in PSA from an emergency perspective compared to ketamine used alone. According to the analysis, ketofol is significantly preferable in relation to most aspects. According to the prospective case series conducted by Andolfatto and Willman, ketofol has a significantly short recovery time compared to single agents, including ketamine [9]. In the study, the median recovery time for ketofol was 14 minutes for PSA (ranging from three to 50 minutes on average) (recovery in less than 20 minutes occurred in 90% of the participants) [9]. Despite the fact that recent trials based on the intensive care unit setting or in the operating room have described ketofol use as advantageous with regard to fastidious recovery and hemodynamic stability, articles documenting ketofol use in the emergency department setting are quite limited [10,11].

According to the findings of the meta-analysis (Figure 4), ketofol showed minimal or no impact on clinician satisfaction. The same case was found to apply to respiratory adverse events, including respiratory depression, airway obstruction, desaturation, and apnea, when compared to ketamine. In the case of other adverse events such as cardiovascular effects, ketofol use significantly reduced the hypotension frequency in patients, but there were no implications on bradycardia. Additionally, as shown by the meta-analysis depicted in Figure 3 and Figure 4, there was no significant difference in the adverse events associated with gastrointestinal aspects such as vomiting and nausea when ketofol or ketamine was used [6,12,13].

The potential equilibrium of implications of ketamine and propofol has led some scientists to favor the combination overuse of either of the agents solely. Despite the fact that the optimum ratio and dosage of both propofol and ketamine from an emergency department perspective for PSA has not yet been fully elucidated and defined, the studies included in the current systematic review and meta-analysis suggest that the titration of a one-syringe combination of propofol and ketamine in a ratio of 1:1 can potentially provide deep sedation with a low probability of the incidence of adverse effects and some significant merits such as short periods of recovery, in spite of their variances in action mechanics and kinetics. The diversity of procedural treatments that medical personnel must perform in the emergency department under PSA and the wide array of responses to both analgesics and sedatives shown by patients results in different requirements for sedatives and analgesics that may be challenging to forecast. Similar-syringe titration of propofol and ketamine may be a method of addressing the aforementioned diverse requirements for analgesia and sedation in an efficient and effective fashion.

The rates of the adverse event recorded by the study by Weisz et al. [13] were much higher than those reported in similar trials conducted earlier by Andolfatto and Willman (2011) [9] and Shah et al. (2011) [12]. The difference may be attributed to the fact that the study included a very highly trained research associate who documented any adverse events, including minimal signs during the PSA. The adverse implications were documented more regularly when observed by an autonomous party rather than conveyed by healthcare providers caring for the participants [14].

According to Aboeldahab et al. (2011) [15], ketofol is a safe and efficient alternative agent that does not have numerous extreme side effects of its two constituents [9]. The findings of all the studies supported the fact that ketofol is an effective PSA agent in patients in the emergency department. The period taken for recovery was quite short, and very few adverse events were recorded. Additionally, the participants and emergency department staff reported that they were highly satisfied.

Limitations

In this systematic review and meta-analysis, efforts were applied to control and restrict the specific variables that would be applied for analysis in the Review Manager. The limitations were applied through a sensitivity examination of all the studies that passed the inclusion criteria. There is a considerable probability of bias due to diverse intrinsic differences between particular studies that focus on different aspects. In some cases, the studies failed to provide data on between-group testing and concentrate on within-group testing. Another restraint is that, in some cases, the participants, therapists, and assessors were not blinded; therefore, there is a possibility of a selection bias in relation to the data collected. Subgroup analysis was not possible in some cases since they measured different adverse events. For instance, the study by Aboeldahab et al. (2011) [15] measured the mean arterial pressure and heart rates.

The limitations also included a language restraint; therefore, other viable studies were excluded. The most impactful restraint is the fact that the eligibility criteria included only studies with more than 50 participants. All the limitations negatively affected the reliability of the findings and the general quality of the systematic review and meta-analysis since some studies that would have been viable for the research had to be excluded.

## Conclusions

No conflicting results were associated with this systematic review and meta-analysis, the findings aligned with the previous studies, and there were only a few differences in relation to rates of adverse events. Fortunately, all the randomized controlled studies were of high quality. Considering the high quality of the studies included in the systematic review and meta-analysis, the evidence from the studies is a true projection of the actual efficiency of ketofol when compared to ketamine for PSA from an emergency perspective. There were a few adverse effects reported in the studies, including nausea and vomiting.

According to the findings from the systematic review and meta-analysis, ketofol has a significantly short recovery time compared to single agents, including ketamine. Ketofol also showed minimal to no impact on clinician satisfaction and did not result in respiratory adverse events compared to ketamine. The ketofol and ketamine groups were also found to have similar associations between the levels of sedation and the BIS values. The time required for verbal contact loss and the reflex of eyelashes was found to be much earlier in the ketofol group in comparison to the ketamine group. There was no significant difference in type/nature or extent of the adverse events, excluding nausea, which was found to be more prevalent in the ketamine group. Therefore, ketofol was found to be safe and more effective in comparison to ketamine for PSA from an emergency perspective. Future studies should look into the ratio of the combination of propofol and ketamine for ketofol.
